# Proteome Size Is Positively Correlated with Lifespan in Mammals but Negatively Correlated with Lifespan in Birds

**DOI:** 10.1002/adbi.202400633

**Published:** 2025-02-17

**Authors:** Juliano Morimoto, Zuzanna Pietras

**Affiliations:** ^1^ Institute of Mathematics School of Natural and Computing Sciences University of Aberdeen Fraser Noble Building Aberdeen AB24 3UE UK; ^2^ Programa de Pós‐graduação em Ecologia e Conservação Universidade Federal do Paraná Curitiba 82590‐300 Brazil; ^3^ Department of Physics Chemistry and Biology (IFM) Linköping University Linköping 581 83 Sweden

**Keywords:** comparative genomics, dietary restriction, trade‐offs

## Abstract

The central dogma describes the unidirectional flow of genetic information from DNA to proteins, leading to an underappreciation of the potential for the information contained in proteomes (the full set of proteins in an organism) to reflect broader biological processes such as lifespan. Here, this is addressed by examining how the size and composition of 276 proteomes from four vertebrate classes are related to lifespan. After accounting for the relationship between body weight and lifespan, lifespan is negatively correlated with proteome size in birds and, to a weaker extent, in fish, and positively correlated with lifespan in mammals. Proteome composition varies amongst the four vertebrate classes, but there is no evidence that any specific amino acid correlated with lifespan. The findings in relation to the role of dietary amino acid restriction are discussed on lifespan extension and raise questions about evolutionary and structural forces shaping proteome composition across species.

## Graphical abstract

The relationship between proteome size, amino acid composition, and lifespan across 276 vertebrate species is investigated. Proteome size correlates positively with lifespan in mammals but negatively in birds and fish. While proteome composition varies among classes, no single amino acid frequency correlates with lifespan. The findings highlight how proteome data can help uncover knowledge about life history trait evolution across the tree of life.

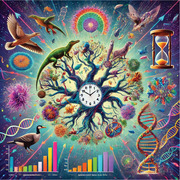

## 1 Introduction

The central dogma describes a unidirectional flow of information from DNA to proteins as a result of the degenerate genetic code.^[^ ^1^, ^2^ ^]^ We now know that both the genome and the proteome (defined here as the collection of all proteins of a genome) of species contain valuable information on species’ evolutionary history, adaptations, patterns of selection, and metabolic trade‐offs.^[^ ^3^, ^4^, ^5^, ^6^ ^]^ With the growing accessibility of genome‐sequencing technologies, we are only at the dawn of discoveries that will uncover novel patterns encoded in the genome and proteome across the tree of life.^[^ ^7^, ^8^ ^]^ Yet, many areas of genome science remain unexplored, including studies examining the proteome for signatures of broader biological processes affecting Darwinian fitness.

The genome contains information about broader biological processes such as lifespan, as is the case, for example, for the density of CpG sites in gene promoters.^[^ ^9^, ^10^ ^]^ But given that the genetic code degenerates, can we even expect any correlation between biological processes to persist at the proteome level? The relatively smaller literature that focused on proteomes – specifically in terms of the amino acid usage in proteomes (sometimes referred to as the “exome”) – suggests that proteomes contain valuable insights. For example, optimal growth temperature is associated with relatively lower frequencies of the thermolabile amino acid cysteine in the proteomes of unicellular organisms.^[^ ^3^, ^11^, ^12^ ^]^ We also contributed to novel insights by uncovering a novel edge effect across proteomes, whereby only a few amino acids are unusually used across genomes.^[^ ^13^ ^]^ Moreover, amino acid usage in the proteome contains species‐specific information on the amino acid requirements to optimize growth and reproduction without lasting effects on lifespan in flies and mice.^[^ ^14^, ^15^ ^]^ However, we still lack a proper test as to whether the proteome contains information about lifespan in taxonomically diverse datasets. Specifically, we lack an understanding of how proteomes vary across major vertebrate groups, which poses a significant challenge to characterizing how the proteome influences life histories in complex multicellular species. Without this knowledge, we cannot properly ascertain whether proteomic patterns can be detected at broader scales across the tree of life.

Here, we addressed this knowledge gap by analyzing for the first time the amino acid usage of 276 species of vertebrates for which phylogeny, as well as maximum lifespan and adult body weight data, are available in the literature.^[^ ^16^, ^17^ ^]^ We used maximum lifespan because it is one of the easiest and most complete sets of life history information in our database. This can pose challenges for interpretation in relation to aging and senescence because maximum lifespan does not necessarily correlate with senescence rates.^[^ ^18^ ^]^ Nevertheless, maximum lifespan is an important life history trait as it is involved in demographic models, and, from a human perspective, it is an important health outcome that has received theoretical and empirical attention.^[^ ^19^, ^20^, ^21^, ^22^, ^23^, ^24^ ^]^ Our main goals were to (1) test whether proteome size correlated with lifespan, (2) test whether proteome composition differed between major vertebrate groups and (3) ascertain whether the frequencies of specific amino acids in the proteome of different groups were positively or negatively correlated with lifespans.

## 2 Experimental Section

### Data and Approach

We retrieved translated coding sequences FASTA files from the FTP server in NCBI, downloading only reference sequence (RefSeq) genomes to ensure maximum coverage and annotation accuracy. Amino acid proportions were calculated as the sum of each amino acid in the genome divided by the total amino acid count, as described in previous work.^[^ ^13^, ^14^ ^]^ We assembled an amino acid usage frequencies database by calculating the relative frequency of each of the 20 amino acids as a proportion of the total amino acid counts across 286 species using coding sequence files from the NCBI server (accessed before 01‐January‐2024, Data S1). Only RefSeq genome proteome data with associated taxonomy were considered.^[^ ^13^ ^]^ Maximum lifespan information and average adult body weight (in grams) were collected from the AnAge database (accessed: 20‐March‐2024).^[^ ^16^ ^]^ Notably, the AnAge database does not specify the sex of individuals from which lifespan and body weight data were collected. For most animal species, especially vertebrates, there can be substantial sexual dimorphisms.^[^ ^25^, ^26^, ^27^ ^]^ This limitation should be addressed in future database releases. All analyses were conducted in R 4.3.2 (R Core Team, 2023).

**Figure 1:**
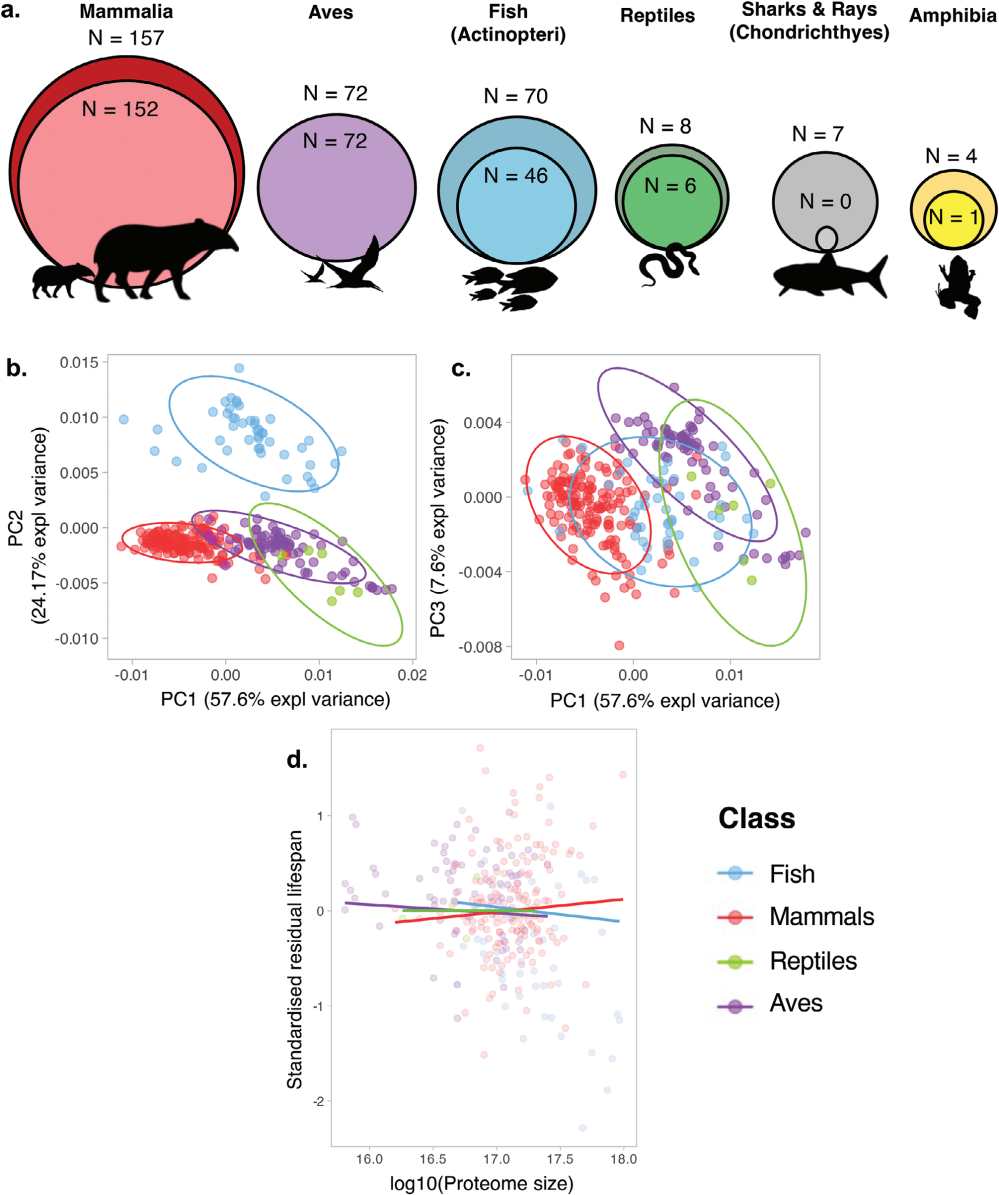
The relationship between proteome size and composition and lifespan across vertebrates. a) Initially, we had life history (lifespan and body weight) data for seven classes and 318 species, but proteome data were acquired for only 286 species, distributed across six classes (larger circles). Of these, 276 species, distributed across four classes, had complete phylogenetic data (smaller circles). Note that amphibians were excluded from the analysis because only one species had complete information (i.e., proteome, lifespan, body weight, and phylogenetic placement). b,c) PCA on the 276 species with established phylogenetic placement, which were used for the comparative analysis. d) The relationship between proteome size (log10‐transformed) and standardized residual lifespan.

For all regression analyses, we controlled for non‐independent observations in our dataset arising from evolutionary (phylogenetic) relationships between species. For instance, species that are more closely related evolutionarily may exhibit more similar amino acid profiles, body weight, and lifespans. Failing to account for this non‐independence could result in artifacts.^[^ ^27^ ^]^ To address this, we used phylogenetically controlled linear regressions (PGLS) to account for potential non‐independence in our data. Phylogenetic relationships were obtained from megatrees accessed using the rtrees package [see [17] and references therein]. Our analyses focused on major vertebrate classes, utilizing data on maximum lifespan or adult body weight (or both) for 318 species across six major vertebrate classes. Proteome data were available for 286 of these species, including Mammalia (157), Aves (72), Fish (70), Amphibia (4), Reptilia (8), and Chondrichthyes (7). However, not all species had complete phylogenetic relationship data. Consequently, our final analyses included 276 species across five classes: Mammalia (152), Aves (72), Fish (46), Amphibia (1), and Reptilia (6) (**Figure** **1**a). Amphibia was excluded from downstream regression analyses due to insufficient data. All proteome analyses used PGLS models with a Brownian correlation structure, implemented using the gls function from the nlme 3.1‐164 package.^[^ ^28^ ^]^ We also controlled for the confounding effects of adult body weight on lifespan. To do so, we first fitted a PGLS of log10‐maximum lifespan against log10‐square‐root‐body weight. The relationship between these transformed variables was isometric (Table S1, Supporting Information). Residuals from this regression were extracted and used as the response variable, referred to as residual standardized lifespan, for all subsequent analyses.

We tested whether proteome composition differed across vertebrate classes. Analyses were performed for all 286 species with available proteome data and either maximum lifespan or adult body weight information, as well as the 276 species with proteome, life‐history, and phylogeny data (Figure S1, Supporting Information). To investigate this, we conducted a principal component analysis (PCA) using the prcomp() function and performed PERMANOVA using the adonis2 function from the vegan package v2‐6.4^[^ ^29^, ^30^ ^]^ to assess the statistical significance of vertebrate class effects on PC1–PC3, which explained more than 85% of the variance in the data. The remaining variance was accounted for by the other 17 PCs.

Next, we tested whether proteome size (i.e., the total number of amino acids in the proteome) correlated with residual standardized lifespan. We fitted a PGLS with residual standardized lifespan as the dependent variable and log‐transformed proteome size as the independent variable. Additionally, we investigated correlations between the frequencies of the 20 individual amino acids and residual standardized lifespan. Amino acid frequencies were normalized using the scale() function with default parameters in base R. Statistical significance of regression models was assessed using the confint() function to calculate confidence intervals (CIs) for the slopes of each regression (**Table** **1**). For all PGLS regressions, the p‐value significance level was set to 0.0042 to account for multiple comparisons. Similarly, significance levels for confidence intervals on individual amino acids were set to 99.75% to account for the 20 regressions (one for each amino acid) performed per class. Statistical significance was determined when the CIs did not overlap zero. Due to the need to account for phylogenetic signal and the absence of a single unifying tree, separate PGLS models were fitted for each major vertebrate class.

**Table 1:** Slope and confidence interval of the slope between amino acid frequency in the proteome and standardized residual lifespan, per vertebrate class. Lwr: lower confidence interval; Upr: upper confidence interval; slope: an estimate of the slope from the PGLS.

	Mammals			Aves			Fish			Reptiles		
Amino acid	lwr	slope	upr	lwr	slope	upr	lwr	Slope	upr	lwr	slope	upr
Alanine	−0.124	−0.024	0.075	−0.087	0.074	0.234	−0.598	−0.013	0.573	−0.181	0.122	0.424
Arginine	−0.144	−0.037	0.071	−0.099	0.061	0.222	−0.132	0.368	0.867	−0.397	−0.041	0.315
Asparagine	−0.084	0.012	0.107	−0.271	−0.104	0.063	−0.782	−0.178	0.425	−0.463	−0.142	0.178
Aspartate	−0.034	0.046	0.126	−0.218	−0.065	0.088	−0.520	−0.208	0.104	−0.178	0.063	0.304
Cysteine	−0.076	0.004	0.084	−0.212	−0.017	0.177	−0.116	0.115	0.346	−0.273	0.006	0.284
Glutamate	−0.081	−0.009	0.063	−0.284	−0.091	0.103	−0.328	−0.059	0.210	−0.143	0.052	0.247
Glutamine	−0.129	−0.048	0.033	−0.208	−0.015	0.177	−0.065	0.175	0.415	−0.232	0.097	0.427
Glycine	−0.083	0.009	0.101	−0.046	0.112	0.269	−0.582	0.083	0.747	−0.164	0.098	0.361
Histidine	−0.130	−0.045	0.040	−0.183	−0.008	0.168	−0.159	0.244	0.647	−0.295	−0.015	0.264
Isoleucine	−0.065	0.039	0.144	−0.270	−0.093	0.084	−0.881	−0.366	0.150	−0.520	−0.110	0.301
Leucine	−0.049	0.027	0.103	−0.267	−0.105	0.058	−0.076	0.139	0.353	−0.332	0.046	0.425
Lysine	−0.085	0.007	0.098	−0.229	−0.076	0.077	−0.756	−0.342	0.073	−0.374	−0.040	0.294
Methionine	−0.081	0.005	0.092	−0.284	−0.096	0.092	−0.119	0.255	0.628	−0.283	−0.012	0.259
Phenylalanine	−0.064	0.007	0.079	−0.264	−0.090	0.084	−0.202	0.088	0.377	−0.299	0.037	0.372
Proline	−0.124	−0.033	0.058	−0.043	0.119	0.280	−0.472	0.031	0.533	−0.447	−0.081	0.285
Serine	−0.134	−0.035	0.063	−0.222	−0.004	0.215	−0.316	0.043	0.401	−0.166	−0.052	0.062
Threonine	−0.074	0.023	0.120	−0.128	0.042	0.213	−0.477	−0.174	0.128	−0.286	−0.074	0.138
Tryptophan	−0.033	0.056	0.145	−0.177	0.022	0.220	−0.252	−0.022	0.209	−0.214	−0.073	0.068
Tyrosine	−0.081	0.005	0.091	−0.277	−0.073	0.132	−0.294	0.000	0.295	−0.356	0.071	0.498
Valine	−0.039	0.037	0.112	−0.206	−0.003	0.200	−0.291	−0.084	0.122	−0.157	0.071	0.299

## 3 Results

Maximum lifespan was positively correlated with adult body weight across all vertebrate classes (Mammalia = slope: 0.302, std error: 0.007, p < 0.001; Aves = slope: 0.340, std error: 0.015, p < 0.001; Fish = slope: 0.281, std error: 0.032, p < 0.001; Reptilia = slope: 0.219, std error: 0.020, p < 0.001). We then used the residuals from the PGLS regression of lifespan and adult body weight, which effectively controlled for the correlation between lifespan and body weight (Table S1, Supporting Information).

Next, we tested whether proteome size also correlated positively with residual standardized lifespan across each major vertebrate class. Given the well‐known positive correlation between genome size and lifespan in animals, we hypothesized that a similar pattern might hold for proteome size.[^[^ ^31^, ^32^, ^33^ ^]^, but see also^[^ ^34^ ^]^] We found that proteome size was positively correlated with residual standardized lifespan in mammals (slope: 0.135, std error: 0.024, p < 0.001), but negatively correlated in birds (slope: ‐0.088, std error: 0.028, p = 0.0022) and fish (slope: ‐0.155, std error: 0.071, p = 0.029). However, the slope for fish was not statistically significant at the threshold level of 0.0042, which was used to correct for multiple comparisons.

Our PCA analysis revealed differences in the underlying proteome composition profiles across major vertebrate classes (PERMANOVA: F_3272_ = 166.11, p < 0.001). The mammalian proteome profile differed from those of reptiles and, to a lesser extent, birds, primarily in relation to PC1, which had Proline and Lysine as the lowest and highest loadings, respectively (Table S2, Supporting Information). Fish exhibited a unique proteome signature compared to other vertebrate classes in our dataset, primarily differentiated by PC2, with the highest and lowest loadings for Threonine and Leucine, respectively. There was less differentiation between vertebrate classes in relation to PC3, although mammalian proteomes did not overlap with those from reptiles and birds; PC3 had the highest and lowest loadings for Glutamate and Leucine, respectively (Figure 1b,c, see also Figure S1, Supporting Information).

Based on compositional changes in the proteome across classes, we examined whether the frequency of any individual amino acid in the proteome correlated with lifespan. To investigate this, we analyzed the correlation between the frequency of each amino acid and the residual lifespan in each vertebrate class. After controlling for multiple comparisons, our confidence intervals showed no evidence of statistically significant relationships between specific amino acids and residual lifespan (Table 1). This suggests that no single amino acid frequency correlated with lifespan. Together, our results demonstrate that, after controlling for body weight, lifespan negatively correlates with proteome size in birds and, to a lesser extent, in fish, but positively correlates with proteome size in mammals. While there are compositional changes in proteome profiles, these do not translate into direct associations between amino acid frequency and lifespan.

## 4 Discussion

The genome contains valuable information across a wide range of species traits, including life histories.^[^ ^34^, ^35^, ^36^ ^]^ One aspect of genome biology that remains relatively underexplored is the proteome and how the information contained in it can be used to better understand the biology and evolution of animals across the tree of life. In this study, we addressed this gap by investigating the correlation between proteome size and amino acid frequencies in the proteome with the lifespan of 276 species across four major classes of vertebrate animals. Our data showed that, after controlling for body weight, maximum lifespan was negatively correlated with proteome size in birds and, to a smaller extent, in fish, but positively correlated with lifespan in mammals. To our knowledge, this is the first time this has been reported, and we therefore have no available evidence to conjecture the underlying mechanisms. Mammalian and avian genomes exhibit specific adaptive dynamics that modulate genome size, particularly in relation to transposable elements.^[^ ^37^ ^]^ We have yet to learn more about the dynamics and constraints at the proteome level that will provide the necessary mechanistic insights to help explain why proteome size in some classes is positively or negatively correlated with residual lifespan.

We also found that, although the proteome compositional profile differed among classes, it did not correspond to any single individual amino acid frequency correlating with residual lifespan in any of the four classes. We cannot rule out that this lack of statistical significance for the correlations between individual amino acid frequencies in the proteome and lifespan is due to relatively smaller sample sizes, particularly for reptiles (n = 6). However, this is unlikely to explain the patterns for other vertebrate classes for which we had larger sample sizes, such as fish (46), birds (72), and mammals (152). A possible explanation for the lack of relationship between specific amino acids and lifespan is that amino acid frequencies in the proteome are shaped by evolutionary and structural constraints at the molecular level, as opposed to constraints related to life histories such as lifespan (Seligmann, 2003). Thus, the proteome is less dynamic than the amino acid needs of organisms at any given time.

We know that amino acid intake can influence the expression of life histories in a range of animals. For instance, it is known that restricting specific amino acids, particularly (but not only) branched‐chain amino acids (BCAAs), extends lifespan across a wide range of animal taxa (Trautman et al., 2022). Evidence from model organisms suggests that isoleucine restriction increases lifespan, and health, and improves responses to stress.^[^ ^38^, ^39^, ^40^, ^41^ ^]^ Other amino acids also contribute to lifespan modulation based on their intake. Dietary glycine supplementation can improve health and lifespan in mice.^[^ ^42^ ^]^ There have been reports that tryptophan restriction extends lifespan in mice, but more recent studies have shown that threonine supplementation increases *Caenorhabditis elegans* lifespan see [43, 44] and references therein. *Drosophila melanogaster* fed arginine at 20 mm experienced shorter lifespans, with reductions of 16.3% and 21.2% for males and females, respectively.^[^ ^45^ ^]^ Meanwhile, a recent study has shown that restricting lysine and two other essential amino acids extends *D. melanogaster* lifespan to a similar magnitude as the restriction of BCAAs (leucine, isoleucine, valine).^[^ ^46^ ^]^

It is not surprising that amino acid frequency at the proteome level differs from the dynamic responses to amino acid intake. Not all genes are equally expressed all the time, which makes amino acid frequencies at the proteome level a relatively coarse approximation of what species and individuals need at any given time. Yet, proteome‐level amino acid frequencies are remarkably good predictors of dietary needs in both *D. melanogaster* and mice.^[^ ^14^, ^15^, ^47^ ^]^ Moreover, proteome‐level amino acid frequencies are better indicators of dietary needs for reproduction in flies, despite large differences in gene expression (≈50% of genes) between males and females. As a result, flies fed transcriptome‐weighted amino acid diets were worse off compared to those fed proteome‐matched amino acid diets.^[^ ^14^ ^]^ This suggests that amino acid frequencies at the proteome level contain valuable information about the nutritional needs for life histories, independent of fluctuations in gene expression. Future studies should focus on understanding how proteome‐level amino acid frequencies relate to fluctuations in gene expression and dietary needs.

## 5 Conclusion 

We analyzed the amino acid frequencies across the proteomes of four major classes of vertebrate animals and found that proteome size is positively correlated with lifespan in mammals, but negatively correlated with lifespan in birds and, to a weaker extent, fish. There were differences in amino acid frequency in the proteomes of the four classes, but no single amino acid frequency was associated with lifespan in any of the classes. To our knowledge, this is the first comprehensive comparative study of proteome composition and lifespan. With the growing availability of annotated genomic and proteomic data, future studies should explore how molecular information correlates with ecological and organismal traits. This will help us identify biomarkers at the proteome level that can enhance our understanding and prediction of how animals interact with their environment and how the environment shapes their genomes.^[^ ^13^ ^]^

## Conflict of Interest

The authors have no conflict of interests to declare.

## Authors’ contributions

J.M. and Z.P. conceived the study. J.M. and Z.P. analyzed and interpreted the data. J.M. plotted the data and wrote the first draft of the manuscript. Both authors contributed to the revisions.

## Supporting information



Supporting Information

## Data Availability

Data and code are available in GitHub: https://github.com/jmor2753/advbiol_2025_proteome.
