# Supplementary material for: Proteome Size Is Positively Correlated with Lifespan in Mammals but Negatively Correlated with Lifespan in Birds
Source: Adv Biol (Weinh). 2025 Feb 17;9(4):2400633. doi: 10.1002/adbi.202400633 (PMC12001000; doi:10.1002/adbi.202400633)
Supplement: Supplementary file 1 — Supporting Information [file ADBI-9-2400633-s001.docx]

**Supplementary Material: “Proteome size is positively correlated with lifespan in mammals but negatively correlated with lifespan in birds”**

Authorship list: Juliano Morimoto^1,2*^, Zuzanna Pietras^3^

Authors’ Affiliations:

^1^ Institute of Mathematics, School of Natural and Computing Sciences, University of Aberdeen, Fraser Noble Building, Aberdeen, UK AB24 3UE

^2^ Programa de Pós-graduação em Ecologia e Conservação, Universidade Federal do Paraná, Curitiba, 82590-300, Brazil

^3^ Department of Physics, Chemistry and Biology (IFM), Linköping University, Sweden

**Contact information**

Dr Juliano Morimoto

Institute of Mathematics, University of Aberdeen

Fraser Noble Building, AB24 3UE

*Correspondence: [juliano.morimoto@abdn.ac.uk](mailto:juliano.morimoto@abdn.ac.uk)

**Figure S1.** (a-b) Principal component analysis (PCA) on the 286 species with proteome data available (a) PC1 and PC2 and (b) PC1 and PC3.

**Table S1. Complete output of PGLS models.**

| **Mammals** | | | | | **Aves** | | | | **Fish** | | | | **Reptilia** | | | |
| --- | --- | --- | --- | --- | --- | --- | --- | --- | --- | --- | --- | --- | --- | --- | --- | --- |
| **log(lifespan) ~ log(sqrt(weight)** | | | | | **log(lifespan) ~ log(sqrt(weight)** | | | | **log(lifespan) ~ log(sqrt(weight)** | | | | **log(lifespan) ~ log(sqrt(weight)** | | | |
|  | *Value* | *s.e.m* | *t-value* | *p* | *Value* | *s.e.m* | *t-value* | *p* | *Value* | *s.e.m* | *t-value* | *p* | *Value* | *s.e.m* | *t-value* | *p* |
| (Intercept) | 1.705 | 0.049 | 34.535 | **<0.001** | 1.787 | 0.074 | 24.318 | **<0.001** | 2.225 | 0.166 | 13.428 | **<0.001** | 2.168 | 0.033 | 65.371 | **<0.001** |
| log(weight^0.5) | 0.303 | 0.007 | 43.399 | **<0.001** | 0.341 | 0.016 | 21.871 | **<0.001** | 0.281 | 0.032 | 8.653 | **<0.001** | 0.219 | 0.020 | 10.701 | **<0.001** |
| **Residual lifespan ~ log(sqrt(weight)** | | | | | **Residual lifespan ~ log(sqrt(weight)** | | | | **Residual lifespan ~ log(sqrt(weight)** | | | | **Residual lifespan ~ log(sqrt(weight)** | | | |
| (Intercept) | -3.75E-16 | 4.94E-02 | -7.60E-15 | 1 | 3.84E-16 | 7.35E-02 | 5.22E-15 | 1 | 1.64E-15 | 1.66E-01 | 9.91E-15 | 1 | -1.68E-16 | 3.32E-02 | -5.08E-15 | 1 |
| log(weight^0.5) | 1.04E-17 | 6.98E-03 | 1.49E-15 | 1 | -1.42E-16 | 1.56E-02 | -9.09E-15 | 1 | -1.24E-16 | 3.25E-02 | -3.82E-15 | 1 | -8.01E-17 | 2.05E-02 | -3.91E-15 | 1 |
| **Residual lifespan ~ log(proteome size)** | | | | | **Residual lifespan ~ log(proteome size)** | | | | **Residual lifespan ~ log(proteome size)** | | | | **Residual lifespan ~ log(proteome size)** | | | |
| (Intercept) | -2.318 | 0.426 | -5.439 | **<0.001** | 1.481 | 0.486 | 3.048 | **0.002** | 2.676 | 1.228 | 2.178 | **0.030** | 0.100 | 0.949 | 0.105 | 0.917 |
| log(proteome_size) | 0.136 | 0.025 | 5.460 | **<0.001** | -0.089 | 0.029 | -3.064 | **0.002** | -0.155 | 0.071 | -2.184 | **0.029** | -0.006 | 0.056 | -0.105 | 0.917 |

**Table S2. PCA loadings.**

| **Amino acid** | **PC1** | **PC2** | **PC3** | **PC4** | **PC5** | **PC6** | **PC7** | **PC8** | **PC9** | **PC10** | **PC11** | **PC12** | **PC13** | **PC14** | **PC15** | **PC16** | **PC17** | **PC18** | **PC19** | **PC20** |
| --- | --- | --- | --- | --- | --- | --- | --- | --- | --- | --- | --- | --- | --- | --- | --- | --- | --- | --- | --- | --- |
| Leucine | -0.226 | -0.420 | -0.423 | -0.199 | -0.327 | -0.135 | 0.324 | 0.130 | -0.020 | -0.191 | 0.336 | -0.214 | 0.002 | -0.129 | -0.113 | 0.096 | 0.146 | 0.017 | 0.042 | -0.224 |
| Lysine | 0.319 | -0.339 | 0.317 | -0.089 | 0.222 | -0.156 | 0.005 | -0.047 | 0.023 | -0.192 | -0.340 | -0.340 | -0.020 | -0.476 | 0.012 | -0.040 | -0.012 | -0.177 | 0.143 | -0.224 |
| Isoleucine | 0.300 | -0.282 | -0.096 | -0.035 | 0.215 | 0.041 | -0.354 | 0.085 | -0.005 | 0.131 | 0.388 | 0.190 | 0.242 | 0.128 | -0.161 | -0.495 | 0.017 | 0.043 | 0.193 | -0.224 |
| Phenylalanine | 0.066 | -0.242 | -0.324 | 0.067 | -0.046 | 0.147 | -0.218 | 0.192 | -0.091 | -0.103 | -0.227 | 0.245 | -0.001 | 0.099 | 0.511 | 0.276 | -0.413 | -0.120 | 0.087 | -0.224 |
| Alanine | -0.347 | -0.233 | 0.438 | 0.461 | -0.343 | 0.067 | -0.229 | -0.291 | 0.187 | 0.038 | 0.206 | -0.061 | 0.108 | -0.016 | 0.087 | 0.053 | -0.072 | -0.001 | 0.034 | -0.224 |
| Glutamate | 0.070 | -0.147 | 0.482 | -0.120 | 0.138 | -0.212 | 0.350 | 0.190 | -0.323 | 0.129 | 0.114 | 0.261 | 0.190 | 0.302 | 0.033 | 0.333 | 0.067 | 0.061 | -0.074 | -0.224 |
| Tryptophan | -0.021 | -0.066 | -0.061 | 0.008 | -0.006 | -0.058 | -0.019 | -0.055 | -0.148 | 0.058 | -0.066 | -0.089 | -0.002 | 0.016 | 0.049 | -0.305 | -0.112 | -0.143 | -0.876 | -0.224 |
| Tyrosine | 0.133 | -0.055 | -0.115 | 0.176 | 0.077 | 0.019 | -0.050 | -0.101 | 0.149 | -0.052 | -0.100 | 0.371 | -0.204 | -0.240 | 0.148 | 0.109 | 0.551 | 0.478 | -0.167 | -0.224 |
| Cysteine | 0.022 | -0.038 | -0.172 | 0.034 | -0.119 | 0.047 | -0.076 | -0.291 | -0.282 | 0.309 | -0.334 | -0.226 | -0.074 | 0.395 | -0.021 | 0.002 | 0.447 | -0.267 | 0.198 | -0.224 |
| Proline | -0.533 | -0.016 | 0.036 | -0.366 | 0.376 | -0.186 | -0.309 | 0.037 | 0.339 | -0.134 | -0.144 | 0.053 | -0.140 | 0.221 | -0.154 | 0.083 | 0.027 | -0.039 | -0.011 | -0.224 |
| Asparagine | 0.316 | -0.009 | 0.030 | 0.051 | 0.092 | 0.342 | 0.001 | 0.057 | 0.167 | 0.130 | 0.205 | -0.315 | -0.489 | 0.199 | -0.276 | 0.308 | -0.221 | 0.156 | -0.096 | -0.224 |
| Glutamine | -0.018 | 0.054 | 0.140 | -0.250 | -0.132 | 0.133 | 0.451 | -0.268 | 0.149 | -0.059 | -0.031 | 0.234 | -0.347 | 0.122 | 0.236 | -0.463 | -0.178 | 0.014 | 0.188 | -0.224 |
| Histidine | -0.017 | 0.083 | -0.147 | -0.113 | -0.023 | 0.080 | 0.148 | -0.197 | 0.134 | 0.186 | -0.323 | -0.161 | 0.546 | 0.012 | -0.143 | 0.031 | -0.266 | 0.517 | 0.024 | -0.224 |
| Arginine | -0.273 | 0.125 | 0.044 | 0.037 | -0.076 | 0.082 | 0.021 | 0.496 | -0.066 | 0.596 | -0.114 | 0.070 | -0.153 | -0.403 | -0.069 | -0.123 | -0.026 | -0.044 | 0.110 | -0.224 |
| Methionine | 0.106 | 0.155 | -0.119 | 0.097 | 0.029 | 0.208 | 0.204 | -0.155 | 0.263 | -0.059 | 0.004 | 0.383 | 0.233 | -0.158 | -0.368 | 0.207 | 0.023 | -0.548 | -0.054 | -0.224 |
| Valine | 0.071 | 0.209 | -0.104 | 0.364 | -0.147 | -0.574 | -0.040 | 0.007 | -0.207 | -0.214 | -0.124 | 0.136 | -0.214 | 0.065 | -0.364 | -0.080 | -0.256 | 0.106 | 0.130 | -0.224 |
| Glycine | -0.307 | 0.213 | -0.073 | 0.271 | 0.497 | 0.321 | 0.147 | -0.060 | -0.426 | -0.289 | 0.165 | -0.165 | 0.048 | -0.100 | 0.078 | -0.072 | 0.010 | 0.049 | 0.107 | -0.224 |
| Aspartate | 0.134 | 0.294 | 0.073 | 0.195 | -0.122 | -0.005 | 0.092 | 0.513 | 0.368 | -0.257 | -0.062 | -0.259 | 0.201 | 0.270 | 0.208 | -0.163 | 0.239 | -0.061 | -0.001 | -0.224 |
| Serine | 0.078 | 0.321 | 0.199 | -0.450 | -0.412 | 0.243 | -0.372 | 0.016 | -0.316 | -0.289 | 0.049 | 0.046 | 0.043 | -0.140 | -0.094 | 0.067 | 0.067 | 0.065 | -0.018 | -0.224 |
| Threonine | 0.128 | 0.397 | -0.124 | -0.139 | 0.105 | -0.403 | -0.076 | -0.256 | 0.107 | 0.261 | 0.397 | -0.160 | 0.031 | -0.166 | 0.400 | 0.175 | -0.041 | -0.105 | 0.041 | -0.224 |
